# Performance of three rapid diagnostic tests for the detection of *Cryptosporidium* spp*.* and *Giardia duodenalis* in children with severe acute malnutrition and diarrhoea

**DOI:** 10.1186/s40249-019-0609-6

**Published:** 2019-11-28

**Authors:** Joseph Bitilinyu-Bangoh, Wieger Voskuijl, Johnstone Thitiri, Sandra Menting, Nienke Verhaar, Laura Mwalekwa, Daisy B. de Jong, Merlin van Loenen, Petra F. Mens, James A. Berkley, Robert H. J. Bandsma, Henk D. F. H. Schallig

**Affiliations:** 1 Academic Medical Centre, Department of Medical Microbiology, Parasitology Unit, Amsterdam University Medical Centres, Meibergdreef 9, 1105 AZ Amsterdam, The Netherlands; 2 Queen Elizabeth Central Hospital, Blantyre, Malawi; 3 College of Medicine, Department of Paediatrics and Child Health, University of Malawi, Blantyre, Malawi; 4 Global Child Health Group, Emma Children’s Hospital, Amsterdam University Medical Centres, Amsterdam, The Netherlands; 5 The Childhood Acute Illness and Nutrition Network (CHAIN), Nairobi, Kenya; 6 KEMRI/Wellcome Trust Research Programme, Kilifi, Kenya; 7 Translational Medicine Program, Hospital for Sick Children, Toronto, Canada

**Keywords:** Diagnosis, *Cryptosporidium*, *Giardia*, Rapid diagnostic test, Severe acute malnutrition

## Abstract

**Background:**

There is significant need for accurate diagnostic tools for *Cryptosporidium* spp. and *Giardia duodenalis* infections in resource limited countries where diarrhoeal disease caused by these parasites is often prevalent. The present study assessed the diagnostic performance of three commercially available rapid diagnostic tests (RDTs) based on faecal-antigen detection for *Cryptosporidium* spp. and/or *G. duodenalis* infections in stool samples of children admitted with severe acute malnutrition (SAM) and diarrhoea. An established multiplex PCR was used as reference test.

**Methods:**

Stool samples from children with SAM and diarrhoea enrolled in a randomized controlled trial (registered at clinicaltrials.gov/ct2/show/NCT02246296) in Malawi (*n =* 175) and Kenya (*n =* 120) between December 2014 and December 2015 were analysed by a multiplex PCR for the presence of *Cryptosporidium* spp.*, G. duodenalis* or *Entamoeba histolytica* parasite DNA. *Cryptosporidium-*positive samples were species typed using restriction fragment length polymorphism analysis. A sub-sample of the stool specimens (*n =* 236) was used for testing with three different RDTs. Diagnostic accuracy of the tests under evaluation was assessed using the results of PCR as reference standard using MedCalc software. Pearson Chi-square test and Fisher’s exact test were used to determine (significant) difference between the number of cryptosporidiosis or giardiasis cases found by PCR in Malawi and Kenya. The overall diagnostic accuracy of each RDT was calculated by plotting a receiver operating characteristic (ROC) curve for each test and to determine the area under the curve (AUC) using SPSS8 software.

**Results:**

Prevalence of *Cryptosporidium* spp. by PCR was 20.0 and 21.7% in Malawi and Kenya respectively, mostly *C. hominis. G. duodenalis* prevalence was 23.4 and 5.8% in Malawi and Kenya respectively. *E. histolytica* was not detected by PCR. RDT testing followed the same pattern of prevalence. RDT sensitivities ranged for cryptosporidiosis from 42.9 to 76.9% and for *G. duodenalis* from 48.2 to 85.7%. RDT specificities ranged from 88.4 to 100% for *Cryptosporidium* spp. and from 91.2 to 99.2% for *G. duodenalis* infections. Based on the estimated area under the curve (AUC) values, all tests under evaluation had an acceptable overall diagnostic accuracy (> 0.7), with the exception of one RDT for *Cryptosporidium* spp. in Malawi.

**Conclusions:**

All three RDTs for *Cryptosporidium* spp. and *Giardia duodenalis* evaluated in this study have a moderate sensitivity, but sufficient specificity. The main value of the RDTs is within their rapidness and their usefulness as screening assays in surveys for diarrhoea.

## Multilingual abstracts

Please see Additional file 1 for translations of the abstract into the five official working languages of the United Nations.

## Background

Diarrhoea is the third leading cause of death amongst children under the age of 5 years worldwide, and responsible over an estimated 500 000 infant deaths in 2016, most of which occur in sub-Saharan Africa and South Asia [1, 2]. Diarrhoea and malnutrition are closely linked; diarrhoea often leads to malnutrition, and malnourished children are more prone to develop diarrhoea, a vicious cycle often difficult to interrupt [3]. Over 80% of diarrhoea-associated deaths are attributable to unsafe water, inadequate sanitation, and insufficient hygiene [4].

Most lethal diarrhoea in children under the age of 2 years is estimated to be caused by rotavirus [5], which may soon be controlled by vaccination programs [6, 7]. This is closely followed by *Cryptosporidium* spp.*,* one of the commonest pathogens and at the same time most poorly understood, water-borne parasite in humans [8]. *Cryptosporidium-*associated diarrhoea in early childhood may cause malnutrition, impaired physical and cognitive development, and ultimately death. Response to treatments is variable, and vaccination is not available. Most importantly, this opportunistic parasite is persistent in immunologically compromised individuals infected with HIV as well among severely malnourished children and substantially increases their risk of death [8, 9]. Another important protozoan pathogen is *Giardia duodenalis* which can also lead to diarrhoea. Unlike *Cryptosporidium* spp.*,* drugs are available to effectively treat giardiasis [8].

Timely and accurate diagnosis of intestinal parasites is important to properly manage infected children, in particular vulnerable populations such as severely malnourished children [8]. Detection of disease-causing intestinal parasites is traditionally done by microscopic examination of stool samples. This can lead to wrong diagnostic conclusions and inappropriate patient management, with harmless parasites being interpreted as disease-causing, while life-threatening parasites may not be detected [8]. In addition to this, it is important to understand its epidemiology for effective prevention and to develop effective control measures [9]. There is thus a need to have more sensitive and specific diagnostic tools in place to aid clinical diagnosis and to support control programs.

In recent years, several companies have developed rapid diagnostic tests (RDTs) that are simple to perform, applicable in resource-restricted settings and with a short test time compared to conventional microscopy for the detection of *Cryptosporidium* spp. and *G. duodenalis*. However, as these products often demonstrate varying performances, diagnostic evaluations are needed to assess their accuracy and usefulness [10].

The aim of the present study was to assess the diagnostic performance of three commercially available faecal-antigen assays to detect *Cryptosporidium* spp. and *G. duodenalis* infections in stool samples collected from severe acute malnourished (SAM) children admitted to hospitals in Malawi and Kenya. The RDTs under study were selected on the basis of affordability and availability. An established multiplex real-time polymerase chain reaction (PCR) assay was used as reference standard to assess the diagnostic performance of the RDTs under evaluation. PCR results were also used to determine the prevalence of the *Cryptosporidium* spp. and *G. duodenalis* infections in the study population and restriction fragment length polymorphism analysis was performed to discriminate between *C. hominis* and *C. parvum* infections in the SAM children.

## Methods

### Study design, location and ethic statement

This diagnostic study was conducted within the framework of the “F75 trial”, a multi-centre, randomized, double-blind intervention trial (ClinicalTrials.gov: NCT02246296). Briefly, this study aimed to determine whether stabilization of malnourished children is improved by reducing carbohydrates and removing lactose in F75, the standard milk formula recommended by the WHO [11]. The trial randomized children with complicated SAM to either receive the standard F75 milk or the modified formulation which was iso-caloric but containing more lipids and less carbohydrates. The trial was hosted in two hospitals in Kenya (Kilifi, Mombasa) and one in Malawi (Blantyre) between December 2014 and December 2015 [12]. The study was approved in Malawi by the Malawi College of Medicine Research and Ethics Committee (COMREC nr P.03/14/1540), the KEMRI Scientific & Ethical Review Unit (SSC2799) in Kenya and Oxford Tropical Research Ethics Committee, United Kingdom (58–14). The work was conducted according to guidelines of Good Clinical Practice, which are based on the principles of the Declaration of Helsinki.

### Stool sample collection and storage

For the present study, 295 stool samples were collected from children with SAM and diarrhoea on enrolment of the original study and stored at − 80 °C within 30 min to 1 h after collection. In total 175 samples were collected in Malawi and 120 samples in Kenya. The samples were shipped to the Netherlands under controlled conditions.

### DNA extraction, molecular detection and *Cryptosporidium* species identification

DNA extraction from all stool samples was performed using an EasyMag DNA extraction machine (BioMerieux, France) following a magnetic silica bead/guanidinium thiocyanate binding protocol [13]. A multiplex real-time PCR assay was used for the detection of all relevant human *Cryptosporidium*-species and for the detection of *Entamoeba histolytica* and *Giardia duodenalis* [14]. In brief, a reaction volume of 25 μl with PCR buffer (HotstartTaq master mix; Qiagen), 5 mmol/L MgCl_2_, 3.7 pmol of each *G. duodenalis*-specific primer (forward primer: 5′-GACGGCTCAGGACAACGGTT-3′; reversed primer: 5′-TTGCCAGCGGTGTCCG-3′), 15 pmol of each *C. hominis/parvum*-specific primer (forward primer: 5′- CTTTTTACCAATCACAGAATCATCAGA − 3′; reversed primer: 5′-TGTGTTTGCCAATGCATATGAA -3′), 3.7 pmol of each *E. histolytica*-specific primer (forward primer: 5′-ATTGTCGTGGCATCCTAACTCA-3′; reversed primer: 5′-GCGGACGGCTCATTATAACA-3′), 3.0 pmol of *G. duodenalis-*specific double-labelled probe (FAM-5′-CCCGCGGCGGTCCCTGCTAG-3′-black hole quencher 1), 3.0 pmol of *C. hominis/parvum*-specific double-labelled probe (NED-5′ TCGACTGGTATCCCTATAA- 3′-MGB), 1.5 pmol of *E. histolytica*-specific Minor Groove Binder-TaqMan probe (VIC-5′-TCATTGAATGAATTGGCCATTT-3′-MGB), and 5 μl of the DNA sample. Amplification consisted of 15 min at 95 °C followed by 40 cycles of 15 s at 95 °C, 30 s at 60 °C, and 30 s at 72 °C. Amplification, detection, and data analysis were performed with the BioRad CFX Real Time System C1000 Thermal Cycler (Bio-Rad Laboratories B.V., Veenendaal. The Netherlands).

This multiplex PCR assay was used as the diagnostic reference standard for the study. All collected stool samples (*n =* 295) were analysed with this multiplex assay to detect the parasite species. The molecular analysis was done independently from the RDT analysis and the laboratory personnel was blinded from the diagnostic test results and the origin of the samples.

In order to discriminate the *Cryptosporidium* species in PCR positive stool samples, restriction fragment length polymorphism (RFLP) analysis was performed following the method developed by Coupe and co-workers [15]. Reference strains (*C. parvum, C. hominis* and *C. meleagridis*) were sourced from American Type Culture Collection through an agreement put in place by BEI Resources (USA) and the Academic Medical Centre (The Netherlands). The following approach was followed. A nested PCR was performed, targeting the 18 s rRNA gene. The primary PCR consisted of a 25 μl mix (1× gotag flexibuffer colourless, 20 mmol/L (NH_4_)_2_SO_4_, 0.2 μmol/L forward primer (5′-CTGGTTGATCCTGCCAGTAG-3′), 0.2 μmol/L reverse primer (5′-TAAGGTGCTGAAGGAGTAAGG-3′), 0.01% Tween20, 0.15 mmol/L dNTPs, 2.5 mmol/L MgCl_2_, 1.25 U *Taq* polymerase) combined with 5 μl template DNA. The first PCR program consisted of 39 cycles of annealing at 60 °C for 45 s, extension at 72 °C for 90 s, denaturation at 94 °C for 30 s with an initial denaturation at 94 °C for 5 min and a final extension at 72 °C for 10 min. The secondary PCR consisted of a 45 μl mix (1× gotag flexibuffer colourless, 20 mmol/L (NH_4_)_2_SO_4_, 0.4 μmol/L forward primer (5′-CAGTTATAGTTTACTTGATAATC-3′), 0.4 μmol/L reverse primer (5′-CAATACCCTACCGTCTAAAG-3′), 0.01% Tween20, 0.15 mmol/L dNTPs, 2.5 mmol/L MgCl_2_, 1.25 U *Taq* polymerase) combined with 5 μl PCR1 product. The second PCR program consisted of 39 cycles of annealing at 58 °C for 45 s, extension at 72 °C for 60 s, denaturation at 94 °C for 30 s with an initial denaturation at 94 °C for 5 min and a final extension at 72 °C for 10 min. The resulting *Cryptosporidium-*specific amplicon of 214 bp were subsequently subjected to enzymatic digestion using *Taq*I and *Vsp*I restriction enzymes. The *Taq*I digestion mix (10 μl nested PCR product, 2 units *Taq*I, *Taq*I Buffer) was incubated at 65 °C for 2 h and the *Vsp*I digestion mix (10 μl nested PCR product, 2 units *Vsp*I, 10x Buffer O) at 37 °C for 2 h. Gel-electrophoresis was performed on a 2% agarose gel and visualized using ethidium-Bromide staining under UV light. According to the Coupe typing scheme [15], only *C. hominis* en *C. parvum* will be cut into smaller fragments and none of the othr secies. The *Vsp*I digestion only cuts the *C. hominis* amplicon and thereby allows for discrimination between *C. hominis* and *C. parvum*.

### Rapid diagnostic test kits

Three different commercial kits were evaluated: GIARDIA/CRYPTOSPORIDIUM QUIK-CHEK (Produced by TechLab, USA; Lot: 1016037; expiration date 1 April 2018); RIDA-QUICK for Cryptosporidium/Giardia Combi (Produced by R-Biopharm, Germany; Lot: M52.37; expiration date: May 2018); CRYPTO/GIARDIA DUO-Strip (Produced by Coris Bio Concept, Belgium; Lot: 36464 L1602; expiration date: 15 November 2017).

The assays were conducted at room temperature exclusively on stool samples. All tests were performed according to the manufacturer’s instructions. The diagnostic evaluation was performed on all from the two trial sites in Kenya (*n =* 120) and a selection of samples from the Malawi site (*n =* 116), as not all collected samples from Malawi were of sufficient quantity to perform all three RDT tests. In total 236 stool samples were tested with all three RDTs. The RDTs were read by two independent readers who were blinded from the PCR results and in case of discordance (positive vs negative) a third independent reader was consulted whose conclusion was decisive. Test performance was reported in terms of sensitivity and specificity using the multiplex real-time PCR results as reference standard.

### Statistical analysis

Pearson Chi-square test was used to assess whether a significant difference between the numbers of cryptosporidiosis or giardiasis cases found by PCR was observed in the two countries. The specimen numbers analysed in the present study are relatively low, therefore the statistical outcomes obtained where reanalysed with Fisher’s exact test and confirmed. The results of PCR were used to calculate the sensitivity and specificity of each RDT using MedCalc software (https://www.medcalc.org/calc/diagnostic_test.php). The overall diagnostic accuracy of each RDT was calculated by plotting a receiver operating characteristic (ROC) curve for each test and to determine the area under the curve (AUC) using SPSS8 software (Statistical Package for the Social Sciences, IBM Nederland B.V., Amsterdam, The Netherlands) [16]. In general, an AUC of 0.5 suggests no discrimination (i.e., ability to diagnose patients with and without the disease based on the test result), 0.7 to 0.8 is considered acceptable, 0.8 to 0.9 is considered excellent, and more than 0.9 is considered outstanding [17].

## Results

### Prevalence of intestinal protozoa in the whole study population based on PCR

The prevalence of intestinal protozoa *Cryptosporidium* spp. and *Giardia duodenalis* was determined by analysing stool samples with a multiplex PCR. *Cryptosporidium* spp. was detected in 35 Malawian samples, either as mono-infection (*n =* 31) or as mixed infection with *G. duodenalis* (*n* = 4), resulting in a 20.0% prevalence. The arithmetic mean Ct value of the Malawian PCR positive samples was 31.26 (range: 25.10*–*37.50). *Cryptosporidium* (spp.) infection was also detected in 26 Kenyan samples (25 mono-infections, 1 mixed infection), resulting in a 21.7% prevalence. The arithmetic mean Ct value of the Kenyan PCR positive samples was 30.10 (range: 23.50*–*36.53). The prevalence of *Cryptosporidium* spp. infections was not significantly different (*P* = 0.498) between the two countries.

A *G. duodenalis* infection was detected in 41 samples (23.4% prevalence) from Malawi, either as mono-infection (*n =* 37) or mixed infection (*n =* 4). The arithmetic mean Ct value of the Malawian PCR positive samples for *G. duodenalis* was 30.79 (range: 20.28*–*37.50). Seven stool samples from Kenya were found positive by PCR for *G. duodenalis* infection (5.8% prevalence); mean Ct value of 30.89 (range: 27.36*–*34.95). The number of *G. duodenalis* cases found with PCR was significantly lower (*P* < 0.005) in Kenya compared to that of Malawi.

*Entamoeba histolytica* infections were not detected in any of the stool samples by PCR.

### *Cryptosporidium* species identification

PCR positive samples with sufficient amount of initial DNA were subjected to species identification with RFLP. The majority of *Cryptosporidium* isolates from stool samples from Malawi (*n* = 25; 71.4%), as well as Kenya (*n =* 22; 84.6%), were typed as *C. hominis*. Infections with only *C. parvum* were less abundant for Malawi (*n =* 8; 22.9%) and Kenya (*n =* 3; 11.5%), respectively and there were no mixed *Cryptosporidium* species infections. Species identification could not be done on three stool samples, two from Malawi and one from Kenya, due to low amounts of DNA.

### Sensitivity and specificity of three rapid diagnostic tests

There were 236 stool samples available for the evaluation of the three RDTs. The RDT diagnostic results of *Cryptosporidium* spp. and/or *G. duodenalis* for stool samples from Malawi and Kenya are presented in Table 1. The Kenyan samples yielded more *Cryptosporidium* spp., but fewer *G. duodenalis* infections compared to Malawi. Mixed infections were less prevalent in both countries according to the RDT results. No invalid tests were reported during the evaluation of the RDTs and there were no discrepancies in the diagnostic results reported by the two readers.

**Table 1:** RDT diagnostic results of *Cryptosporidium* spp. and / or *Giardia duodenalis* for stool samples from children under five years of age with from Kenya (*n =* 120) and Malawi (*n =* 116)

		*Cryptosporidium n* (%)	*Giardia* *n* (%)	Mixed infection *n* (%)	Negative *n* (%)
RIDA- QUICK	Kenya	24 (20.0%)	8 (6.7%)	6 (5.0%)	82 (68.3%)
	Malawi	21 (18.1%)	16 (13.8%)	4 (3.5%)	75 (64.7%)
	Total	45 (19.1%)	24 10.2%)	10 (4.2%)	157 (99.5%)
DUO- Strip	Kenya	16 (13.3%)	8 (6.7%)	2 (1.7%)	94 (78.3%)
	Malawi	11 (9.5%)	14 (12.1%)	3 (2.5%)	88 (75.9%)
	Total	27 (11.4%)	22 (9.3%)	5 (2.1%)	182 (77.1%)
QUIK- CHEK	Kenya	19 (15.8%)	7 (5.8%)	1 (0.8%)	93 (77.5%)
	Malawi	14 (12.1%)	23 (19.8%)	2 (1.7%)	77 (66.3%)
	Total	33 (14.0%)	30 (12.7%)	3 (1.3%)	170 (72.0%)

*RDT* Rapid diagnostic test

The in-house multiplex real-time PCR assay for *Cryptosporidium* species was positive in 21 of the 116 stool samples that were used for the evaluation from Malawi, and *G. duodenalis* was detected in 28 samples of the 116 samples from Malawi. The sensitivity and specificity of each RDT under evaluation, using PCR as reference standard, are reported per disease and per country or per disease and both countries together in Table 2. The sensitivity for cryptosporidiosis of the tests under evaluation was rather moderate and ranged from 42.9 to 76.9%. For *G. duodenalis* infection the observed sensitivities were 48.2 to 85.7%. In general, the specificity of all tests was much better, ranging from 88.4 to 100% for *Cryptosporidium* spp., and from 91.2 to 99.2% for *G. duodenalis* infection. Based on the AUC values, all test under evaluation had an acceptable overall diagnostic accuracy (> 0.7), with the exception of the DUO-STRIP test for *Cryptosporidium* in Malawi (AUC = 0.688). Overall, the QUIK-CHEK RDT had the highest AUC for both diseases in both countries.

**Table 2:** Diagnostic performance of three RDTs under evaluation for the detection of *Cryptosporidium* spp. and/or *Giardia duodenalis* infection in stool samples of children under the age of 5 years from Kenya or Malawi

			Sensitivity (95% *CI*)	Specificity (95% *CI*)	AUC	PPV (95% *CI*)	NPV (95% *CI*)
RIDA-	*Cryptosporidium*	Kenya	76.9% (56.4*–*91.0%)	89.4% (81.3*–*94.8%)	0.831	66.7% (51.8*–*78.6%)	93.3% (87.4*–*96.6%)
QUICK		Malawi	66.7% (43.0–85.4%)	88.4% (80.2*–*94.1%)	0.746	56.0% (40.3*–*70.1%)	92.3% (86.7*–*95.7%)
		Both countries	72.3% (57.4*–*84.4%)	88.9% (83.5*–*93.0%)	0.793	61.8% (51.0*–*71.6%)	92.8% (89.0*–*95.4%)
	*Giardia*	Kenya	71.4% (29.0*–*96.4%)	91.2% (84.3*–*95.7%)	0.817	33.3% (19.0*–*51.5%)	98.1% (94.1*–*99.4%)
		Malawi	48.2% (28.7*–*68.1%)	93.3% (85.9*–*97.5%)	0.725	68.4% (47.7*–*83.3%)	85.6% (80.4*–*89.5%)
		Both countries	52.9% (35.1*–*70.2%)	92.1% (87.5*–*95.4%)	0.742	52.9% (39.0*–*66.5%)	92.1% (89.0*–*94.3%)
DUO-	*Cryptosporidium*	Kenya	69.2% (48.2*–*85.7%)	100% (96.2*–*100%)	0.846	100%	92.2% (86.8*–*95.4%)
Strip		Malawi	42.9% (21.8*–*66.0%)	95.8% (89.6*–*98.8%)	0.688	69.2% (43.3*–*86.9%)	88.4% (83.9*–*91.7%)
		Both countries	57.5% (42.2*–*71.7%)	97.9% (94.7*–*99.4%)	0.774	87.1% (71.3*–*94.8%)	90.2% (85.3*–*93.4%)
	*Giardia*	Kenya	57.1% (18.4*–*90.1%)	94.7% (88.8*–*98.0%)	0.759	40.0% (19.6*–*64.6%)	97.3% (93.8*–*98.8%)
		Malawi	51.8% (32.0*–*71.3%)	96.6% (90.5*–*99.3%)	0.742	82.4% (59.2*–*93.8%)	86.9% (81.7*–*90.7%)
		Both countries	52.9% (35.1*–*70.2%)	95.5% (91.7*–*97.4%)	0.742	66.7% (49.5*–*80.3%)	92.3% (89.4*–*94.5%)
QUIK-	*Cryptosporidium*	Kenya	76.9% (56.4*–*91.0%)	100% (96.2*–*100%)	0.885	100%	94.0% (88.6*–*96.9%)
CHEK		Malawi	61.9% (38.4*–*81.9%)	96.8% (91.1*–*99.3%)	0.794	81.3% (57.5*–*93.3%)	92.0% (86.9*–*95.2%)
		Both countries	70.2% (55.1*–*82.7%)	98.4% (95.4*–*99.7%)	0.843	91.7% (77.9*–*97.2%)	93.0% (89.5*–*95.4%)
	*Giardia*	Kenya	85.7% (42.1*–*99.6%)	99.2% (95.2*–*100%)	0.920	85.7% (45.4*–*97.7%)	99.1% (94.8*–*99.9%)
		Malawi	81.5% (61.9*–*93.7%)	96.6% (90.5*–*99.3%)	0.896	88.0% (70.4*–*95.8%)	94.5% (88.6*–*97.4%)
		Both countries	82.4% (65.5*–*93.2%)	98.0% (95.0*–*99.5%)	0.902	87.5% (72.4*–*94.9%)	97.1% (94.1*–*98.6%)

*RDT* Rapid diagnostic test, *95% CI* 95% Confidence interval, *AUC* Area under the curve, *PPV* Positive predictive value, *NPV* Negative predictive value

## Discussion

The present study assessed the diagnostic performance of three commercially available faecal-antigen assays to detect *Cryptosporidium* spp. and/or *G. duodenalis* infections in stool samples using an established multiplex PCR as reference test. Compared to PCR assay, the RDTs have the advantage of being less time-consuming, simpler to carry out, and do not require specialised equipment [18]. All RDTs were valid (no technical test failures) and there were no disagreements between the readings performed by the independent operators, who all reported ease of performance of the RDTs.

All three RDTs tested had a relative low sensitivity, ranging from 48.2% (Malawi) to 85.7% (Kenya) for *G. duodenalis* and from 42.9% (Malawi) to 76.9% (Kenya) for cryptosporidiosis. In contrast, the specificity of the evaluated tests was acceptable (88.4 to 100%). This difference between diagnostic performance, i.e. sensitivity versus specificity, has been previously reported for several RDTs [18–22]. The sensitivities of the three different RDTs employed in the present study was lower than those claimed in the product inserts of the manufacturers (see Table 3). The claimed sensitivities were based on testing stool samples of patients with *Giardia lamblia* or *Cryptosporidium* spp. and comparing the obtained results with microscopy as reference test. In case of Quik Check and Crypto/Giardia Duo-strip, the infecting species (only genus) were not further specified. In case of Rida Quick test the infecting species were according to the product insert *G. lamblia* or *C. parvum.* So there is no evidence presented in the product inserts that the evaluated RDTs can recognise certain rare genetic variants of *G. duodenalis* assemblages (C–F) or less frequent *Cryptosporidium* species (e.g. *C. viatorum, C. ubiquitum, C. cuniculus, C. felis, C. canis*). This could have contributed to the lower sensitivity of the studied RDTs. We do not know however, if these *Cryptosporidium* species or *Giardia* assemblages were present in the patients’ samples as a further genetic characterisation was not performed in the framework of the present study. The lower sensitivity of the RDTs for cryptosporidiosis could in part also be due to the antibodies that are used to detect the parasites are species specific and not genus specific. However, when antibodies have been generated against one particular *Cryptosporidium* species this does not exclude cross-reactivity with other *Cryptosporidium* species. This is confirmed by the observation that all tests are able to detect *C. parvum* as well as *C. hominis* infections. For the other two RDTs this is not the case. The observed lower sensitivity of the RDTs could also be caused by a low parasite density in the faecal samples [18]. In the present study we did not assess the number of parasites in the faecal samples. However, if we take the Ct values as a proxy for parasite load [10], it is noted that there was a huge variety in load in the faecal samples. Stool samples with a Ct value above the mean Ct value were more frequently found negative by RDT, suggesting that there is indeed a sensitivity effect.

**Table 3:** Reported diagnostic performance according to product inserts of three RDTs under evaluation for the detection of *Cryptosporidium* spp. and/or *Giardia* infection in stool samples using microscopy as reference

	Sensitivity	Specificity	Positive predictive value	Negative predictive value
QUIK-CHEK				
*Cryptosporidium* spp.	100%	99.8%	Not reported	Not reported
*Giardia*	98.8%	100%	Not reported	Not reported
RIDA-QUICK				
*Cryptosporidium* spp.	93.8%	100%	100%	97.5%
*Giardia*	100%	95.2%	88.2%	100%
CRYPTO/GIARDIA DUO-Strip				
*Cryptosporidium* spp.	86.7%	100%	100%	97.5%
*Giardia*	89.2%	99.3%	97.1%	97.3%

*RDT* Rapid diagnostic test

In the current study we did not observe major specificity issues. Other studies have reported that RDTs were associated with a false-positive rate of 5–10% [23–25], but the tests used in these studies were from different brands.

The prevalence of *Cryptosporidium* spp. determined by PCR in the stools samples from Kenya (21.7%) is higher than previously described in the review by Squire and Ryan [9] who reported a prevalence range from 3.7 to 9.8% in children under 15 years of age with or without diarrhoea. Other studies also found lower *Cryptosporidium* spp. prevalence in Kenyan children with diarrhoea of 5.1% [26] or 11.0% [27], respectively. In contrast, the prevalence found in the present study was much lower than that previously reported (34.1%) in HIV infected individuals [9]. The HIV status of the participants was not taken into account in the present study. The samples for the present study were collected from SAM cases, and not from children with normal children with “only” diarrhoea. As a consequence of SAM, these children may also have some level of immune suppression which is higher than in normal children, but possibly lower than in HIV infected children.

The *Cryptosporidium* spp. prevalence in the Malawian samples in the present study was 20%, which is approximately two to four times higher than the prevalence (5.9 to 11%) reported in three other studies from the same region [28–30]. The majority of *Cryptosporidium* isolates from Malawi as well as Kenya were typed as *C. hominis* and in line with previous observations [9, 27, 30]. Transmission of the disease is most likely to be from man to man, although the contribution of zoonotic transmission should not be underestimated [30].

The prevalence of *G. duodenalis* in Malawi reported in the present study was 23.4% and is comparable to that found (26%) in a recent study by Huibers et al. [29] in the same setting. A significantly lower prevalence (5.8%) was found with PCR for *G. duodenalis* in Kenya, which is in the range (4.6*–*11%) reported by Squire and Ryan for children ≤15 years with and without diarrhoea [9] or 5.1% prevalence found by Plants-Paris et al. in Kenyan children with diarrhoea [26]. The present study did not perform genotyping (assemblages and subtypes) for *G. duodenalis* and whether this influenced the diagnostic performance of the RDTs*.*

Due to the often moderate diagnostic performance of the RDTs, it is advisable to (if possible within the resources and infrastructure of the setting) to confirm a RDT-positive result with a more sensitive method such as PCR. The big value of the RDTs is within their rapidness and their usefulness as screening assays in large epidemiological surveys. The implementation of RDTs in the health system of resource restricted countries may however be hindered due to the fact that the annual health budget per patient is often very low and the costs of these particular RDTs are relatively high. For the present study we paid in the range from € 6.40/test to € 9.20/test. Possibly a system of subsidized procurement through national Ministries of Health, as for example is in place for malaria RDTs in many disease endemic countries, could alleviate this implementation barrier.

The present study has some limitations. First, the study population comprised children with SAM and diarrhoea. It would be interesting to assess the performance of the RDTs in children with only diarrhoea or even asymptomatic cases. Furthermore, the number of children could be considered low and diagnostic evaluation studies always benefit from a large study population. Finally, the work is restricted to two locations (countries) only, and it is of interest to study other regions, for example countries in south-east Asia, too.

## Conclusions

The sensitivities of the evaluated RDTs are moderate compared to PCR, but their specificity is excellent. Therefore, these RDTs provide a rapid screening method to exclude a *Cryptosporidium* spp. or *G. duodenalis* infection and can provide an alternative diagnostic tool when microscopic examination and technical expertise is unavailable in remote and outbreak settings. This is very helpful in treating vulnerable children in low and middle income countries (LMIC). Next step is to validate these results in non-malnourished children in LMIC and to assess the applicability in a community setting.

## Supplementary information

**Additional file 1.** Multilingual abstracts in the five official working languages of the United Nations.

## Data Availability

The datasets used and/or analysed during the current study are available from the corresponding author on reasonable request.

## Abbreviations

AIDS: Acquired immune deficiency syndrome
ART: Anti-retroviral therapies
AUC: Area under the curve
HIV: Human immunodeficiency virus
LMIC: Low and middle income countries
PCR: Polymerase chain reaction
RDT: Rapid diagnostic test
RFLP: Restriction fragment length polymorphism
ROC: Receiver operating characteristic
SAM: Severe acute malnourished
WHO: World Health Organization

## References

[1] Kotloff KL, Platts-Mills JA, Nasrin D, Roose A, Blackwelder WC, Levine MM (2017). Global burden of diarrheal diseases among children in developing countries: incidence, etiology, and insights from new molecular diagnostic techniques. Vaccine.

[2] Kotloff KL (2017). The burden and ethiology of diarrheal illness in developing countries. Pediatr Clin Nort Am.

[3] Prendergast AJ, Kelly P (2016). Interactions between intestinal pathogens, enteropathy and malnutrition in developing countries. Curr Opin Infect Dis.

[4] Black RE, Morris S, Bryce J (2003). Where and why are 10 million children dying every year?. Lancet..

[5] Clarck A, Black R, Tate J, Roose A, Kotloff K, Lam D (2017). Estimating global, regional and national rotavirus deaths in children aged <5 years: current approaches, new analyses and proposed improvements. PLoS One.

[6] Saha S, Santosham M, Hussain M, Black RE, Saha SK (2018). Rotavirus vaccine will improve child survival by more than just preventing diarrhea: evidence from Bangladesh. Am J Trop Med Hyg..

[7] Lamberti LM, Ashraf S, Walker CL, Black RE (2016). A systematic review of the effect of rotavirus vaccination on diarrhea outcomes among children younger than 5 years. Pediatr Infect Dis J.

[8] Mmbaga BT, Houpt ER (2017). *Cryptosporidium* and *Giardia* infections in children: a review. Pediatr Clin N Am.

[9] Squire SA, Ryan U (2017). *Cryptosporidium* and *Giardia* in Africa: current and future challenges. Parasit Vectors.

[10] Becker SL, Muller I, Mertens P, Herrmann M, Zondie L, Beyleveld L (2017). Acta Trop.

[11] Versloot CJ, Voskuijl W, van Vliet SJ, van de Heuvel M, Carter JC, Phiri A (2017). Effectiveness of three commonly used transition diets in the inpatient management of children with severe acute malnutrition: a pilot randomized controlled trial in Malawi. BMC Pediatr.

[12] Bandsma RHJ, Voskuijl W, Chimwezi E, Fegan G, Briend A, Thitiri J (2019). A reduced-carbohydrate and lactose-free formulation for stabilization among hospitalized children with severe acute malnutrition: a double-blind, randomized control trial. PLoS Med.

[13] Boom R, Sol C, Beld M, Weel J, Goudsmit J, Wertheim-van DP (1999). Improved silica-guanidiniumthiocyanate DNA isolation procedure based on selective binding of bovine alpha-casein to silica particles. J Clin Microbiol.

[14] Bruijnesteijn van Coppenreat LE, Wallinga JA, Ruijs GJ, Bruins MJ, Verweij JJ (2009). Parasitological diagnosis combining an internally controlled real-time PCR assay for the detection of four protozoa in stool samples with a testing algorithm for microscopy. Clin Microbiol Infect.

[15] Coupe S, Sarfati C, Hamane S (2005). Derouin. Detection of *Cryptosporidium* and identification of the species level by nested PCR and restriction fragment length polymorphism. J Clin Microbiol.

[16] Hajian-Tilaki K (2013). Receiver operating characteristic (ROC) curve analysis for medical diagnostic test evaluation. Caspian J Inter Med.

[17] Mandrekar JN (2010). Receiver operating characteristic curve in diagnostic test assessment. J Thor Oncol.

[18] Van de Bossche D, Cnops L, Verschueren VEM (2015). Comparison of four rapid diagnostic tests, ELISA, microscopy and PCR, for the detection of *Giardia lamblia, Cryptosporidium* spp. and *Entamoeba histolytica* in feces. J Microbiol Met.

[19] Minak J, Kabir M, Mahmud I, Liu Y, Liu L, Haque R, Petri WA (2012). Evaluation of rapid point of care tests for the detection of *Giardia* and *Cryptosporidium* species in human fecal specimens. J Clin Microbiol.

[20] Bouyou-Akotet MK, Owondo-Medang M, Moussavou-Boussougou MN, Mabika Mamfoumbi M, Mintsa Nguema R (2016). Low sensitivity of the ImmunocardSTAT® Crypto/Giardia rapid assay test for the detection of *Giardia* and *Cryptosporidium* in fecal samples from children living in Libreville, Central Africa. J Parasit Dis.

[21] Kabir M, Ahmed E, Hossain B, Alam M, Ahmed S, Taniuchi M (2018). Giardia/Cryptosporidium QUIK CHEK assay is more specific than quantitative polymerase chain reaction for rapid point of care diagnosis of cryptosporidiosis in infants in Bangladesh. Clin Infect Dis.

[22] Alexander CL, Niebel M, Jones B (2013). The rapid detection of *Cryptosporidium* and *Giardia* species in clinical samples using the QuikChek immunoassay. Parasitol Int.

[23] Roellig DM, Yoder DS, Madison-Antenucci S, Robinson TJ, Van TT, Collier SA (2017). Community laboratory testing for *Cryptosporidium*: Multicenter study retesting public health surveillance stool samples positive for *Cryptosporidium* by Rapid Cartridge Assay with Direct Fluorescent Antibody Testing. PLoS One.

[24] De Lucio A, Merino FJ, Martinez-Ruiz R, Bailo B, Aquilera M, Fuentes I, Carmena D (2016). Molecular genotyping and sub-genotyping of *Cryptosporidium* spp. isolates from symptomatic individuals attending two major public hospitals in Madrid, Spain. Infect Genet Evol.

[25] De Lucio A, Martinez-Ruiz R, Merino FJ, Bailo B, Aquilera M, Fuentes I, Carmena D (2015). Molecular genotyping of *Giardia duodenalis* isolates from symptomatic individuals attending two major public hospitals in Madrid, Spain. PLoS One.

[26] Plants-Paris K, Bishoff D, Oyaro Mom Mwinyi B, Chappell C, Kituyi A, Nyangao J (2019). Prevalence of *Clostridium difficile* infections among Kenyan children with diarrhea. Int J Infect Dis.

[27] Delahoy MJ, Omore R, Ayers TL, Schilling KA, Blackstock AJ, Ochiengo JB (2018). Clinical, environmental, and behavioral characteristics associated with *Cryptosporidium* infection among children with moderate to severe diarrhea in rural Western Kenya, 2008*–*2012: the global enteric multicenter study (GEMS). PLoS Negl Trop Dis.

[28] Nurminen N, Juuti R, Oikarinen S, Fan YM, Lehto KM, Mangani C (2015). High-throughput multiplex quantitative polymerase chain reaction method for *Giardia lamblia* and *Cryptosporidium* species detection in stool samples. Am J Trop Med Hyg.

[29] Huibers MHW, Moons P, Maseko N, Gushu MB, Iwajomo OH, Heyderman RS (2018). Mulitplex real-time PCR detection of intestinal protozoa in HIV-infected children in Malawi: *Enterocytozoon bieneusi* is common and associated with gastrointestinal complaints and may delay BMI (nutritional status) recovery. Pediatric Inf Dis J.

[30] Morse TD, Nichols RAB, Grimason AM, Campbell BM, Tembo KC, Smith HV (2007). Incidence of cryptosporidiosis species in pediatric patients in Malawi. Epidemiol Infect.

