# Supplementary material for: Performance of three rapid diagnostic tests for the detection of Cryptosporidium spp. and Giardia duodenalis in children with severe acute malnutrition and diarrhoea
Source: Infect Dis Poverty. 2019 Nov 28;8:96. doi: 10.1186/s40249-019-0609-6 (PMC6882336; doi:10.1186/s40249-019-0609-6)
Supplement: Supplementary file 1 — Additional file 1. Multilingual abstracts in the five official working languages of the United Nations. [file 40249_2019_609_MOESM1_ESM.pdf]

Translation of the abstract into the five official working languages of the United Nations

أداء ثلاثة اختبارات تشخيصية للكشف عن داء *خفيات الأبواغ* (كريبتوسبوريديوم) وداء الجيارديا الإثنا عشري (حمى القندس) لدى الأطفال الذين يعانون من سوء التغذية الحاد والإسهال الشديد.

جوزيف بيتيلينيو بانغوه ، ويجر فوسكويل ، جونستون ثيتيري ، ساندرا مينتينج ، نينكي فيرهار ، لورا موالكوا ، ديزي ب. دي يونغ ، ميرلين فان لوينين ، بيترا ف. مينز ، جيمس ه.ج. بيركلي ، روبرت باندسما ، وهينك د.ف.ه. شاليج

ملخص

خلفية: هناك حاجة كبيرة لأدوات التشخيص دقيقة لعدوى داء خفيات الأبواغ (كريبتوسبوريديوم) وعدوى داء الجيارديا الإثنا عشرية في البلدان المحدودة الموارد حيث ينتشر مرض الإسهال الناجم عن هذه الطفيليات. قيمت الدراسة الحالية الأداء التشخيصي لثلاثة اختبارات تشخيصية سريعة ومتاحة تجارياً بناءاً على فحص مستضد عدوى داء خفيات الأبواغ (كريبتوسبوريديوم) في البراز وعدوى داء الجيارديا الإثنا عشرية في عينات البراز من الأطفال الذين يعانون من سوء التغذية الحاد الشديد والإسهال. استُخدم اختبار تفاعل البوليمراز المتسلسل المتعدد الأساسي (Multiplex PCR) كاختبار مرجعي.

الطرق: عينات براز من الأطفال المصابين بنقص التغذية الحاد الشديد والإسهال مسجلين في تجارب مضبوطة عشوائية (مسجلون في clinicaltrials.gov/ct2/show/NCT02246296) في ملاوي (عدد = 175) وكينيا (عدد= 120) بين ديسمبر (كانون الثاني) 2014 وديسمبر (كانون الثاني) 2015 والتي تم تحليلها بواسطة اختبار تفاعل البوليمراز المتسلسل المتعدد الأساسي (Multiplex PCR) لتحري وجود داء خفيات الأبواغ (كريبتوسبوريديوم) وعدوى داء الجيارديا الإثنا عشرية والحمض النووي لطفيلية العدوى الأميبية (Entamoeba histolytica). كانت العينات المصابة بداء خفيات الأبواغ (كريبتوسبوريديوم) هي الأنواع التي تم كشف نوعها باستخدام تحليل تَعَدُّدُ أَشْكَالِ أَطْوالِ الشُّدَفِ. تم استخدام عينة فرعية من عينات البراز (عدد = 236) للفحص وذلك باستخدام ثلاثة أنواع مختلفة من الاختبارات التشخيصية السريعة. تم تقييم دقة التشخيص للاختبارات التي هي قيد التقييم باستخدام نتائج اختبار تفاعل البوليمراز المتسلسل (PCR) كمعيارٍ مرجعي وذلك باستخدام برنامج (MedCalc). وأُستُخدِمَ اختبار مربع تشي لبيرسون واختبار فيشر الدقيق لتحديد الفرق الكبير بين عدد حالات لعدوى داء خفيات الأبواغ (كريبتوسبوريديوم) وعدوى داء الجيارديا الإثنا عشرية التي وجدها اختبار تفاعل البوليمراز المتسلسل (PCR) في ملاوي وكينيا. حُسِبت الدقة التشخيصية الشاملة لكل اختبار تشخيصي سريع (RDT) على حدى من خلال رسم منحنى خاصية تشغيل المستقبل (ROC) لكل اختبار وتحديد المنطقة تحت المنحنى (AUC) باستخدام برنامج (SPSS8).

النتائج: كان انتشار داء خفيات الأبواغ (الكريبتوسبوريديوم) حسب اختبار تفاعل البوليمراز المتسلسل (PCR) بنسبة 0.20٪ و 21.7 ٪ على التوالي في ملاوي وكينيا، ومعظمها من نوع (C. hominis) وكان انتشار داء الجيارديا الإثنا عشرية بنسبة 23.4 ٪ و 5.8 ٪ على التوالي في ملاوي وكينيا. ولم تُرصد أي حالة لطفيلية العدوى الأميبية بواسطة اختبار تفاعل البوليمراز المتسلسل (PCR). اتبع فحص الاختبار التشخيصي السريع مسار الانتشار ذاته. تراوحت حساسيات الاختبار التشخيصي السريع لداء خفيات الأبواغ (كريبتوسبوريديوم) من 42.9 ٪ إلى 76.9 ٪ وأيضاً من 48.2 ٪ إلى 85.7 ٪ لداء الجيارديا الإثنا عشرية. تراوحت خصائص العدوى في الاختبار التشخيصي السريع لداء خفيات الأبواغ (كريبتوسبوريديوم) من 88.4 ٪ إلى 100 ٪ وأيضاً 91.2 ٪ إلى 99.2 ٪ لداء الجيارديا الإثنا عشرية. استناداً إلى المساحة المقدرة الموجودة أسفل قيم المنحنى، كان لجميع الاختبارات قيد التقييم دقة تشخيص مقبولة (< 0.7) ، باستثناء اختبار تشخيصي سريع واحد لداء خفيات الأبواغ (كريبتوسبوريديوم) في مالاوي.

الخاتمة: واجه جميع الاختبارات التشخيصية السريعة (RDTs) لداء خفيات الأبواغ (كريبتوسبوريديوم) وداء الجيارديا الإثنا عشرية التي تم تقييمها في هذه الدراسة حساسية معتدلة ولكن ما يكفي من التخصيص. تتمثل القيمة الرئيسية للاختبارات التشخيصية السريعة في سرعتها وفائدتها كاختبارات فحص في مسوحات الإسهال.

Translated from English version into Arabic by Fadi Moris Kafa, revised by Muhannad Albayk Jaam, through

TRANSLATORS
WITHOUT BORDERS

三种快速诊断隐孢子虫和十二指肠贾第鞭毛虫方法在儿童重症急性营养不良和腹泻中的应用

Joseph Bitilinyu-Bangoh, Wieger Voskuijl, Johnstone Thitiri, Sandra Menting, Nienke Verhaar, Laura Mwalekwa, Daisy B. de Jong, Merlin van Loenen, Petra F. Mens, James A. Berkley, Robert H.J. Bandsma, and Henk D. F. H. Schallig

摘要

引言：在资源有限的国家，由隐孢子虫和十二指肠贾第鞭毛虫等寄生虫引起的腹泻较为普遍，因此迫切需要针对这类感染的准确诊断工具。本研究以建立的多重 PCR 为对照，评估了三种基于粪便抗原检测的商用快速诊断试验(RDTs)对患有重症急性营养不良(SAM)和腹泻的儿童粪便样本中隐孢子虫和/或十二指肠贾第鞭毛虫感染的诊断性能。

方法：2014 年 12 月至 2015 年 12 月在马拉维（n = 175）和肯尼亚（n = 120）进行了一项随机对照试验（在 Clinicaltrials.gov/ct2/show/NCT02246296 注册），收集患有 SAM 和腹泻的患儿粪样。采用多重 PCR 检测隐孢子虫、十二指肠贾第鞭毛虫或溶组织内阿米巴 DNA。使用限制性片段长度多态性分析对隐孢子虫阳性样本进行分型。采用三种不同 RDT 对粪便子样本（n = 236）进行检测。以 PCR 结果为参考标准，使用 MedCalc 软件评估测试的诊断准确性。采用 Pearson 卡方检验和 Fisher 精确检验确定 PCR 发现隐孢子虫病或贾第虫病病例数在马拉维和肯尼亚两地间的差异是否具有统计学意义。通过绘制受试者工作特征（ROC）曲线对每个 RDT 的整体诊断准确性进行评估，并使用 SPSS8 软件计算 ROC 曲线下面积（AUC）。

结果：PCR 检测隐孢子虫在马拉维和肯尼亚的感染率分别为 20.0%和 21.7%，其中以人隐孢子虫为主。十二指肠贾第鞭毛虫在马拉维和肯尼亚的感染率分别为 23.4%和 5.8%。PCR 未检测到溶组织内阿米巴。RDT 检测发现类似的感染模式。RDT 检测隐孢子虫病的敏感性范围为 42.9%～76.9%，十二指肠贾第鞭毛虫为 48.2%～85.7%。RDT 检测隐孢子虫感染的特异性为 88.4%～100%，十二指肠贾第鞭毛虫为 91.2%～99.2%。根据 AUC 值，所有 RDT 均具有可接受的总体诊断准确性(> 0.7)，但其中一种 RDT 对马拉维的隐孢子虫属检测除外。

结论：三种 RDTs 对隐孢子虫和十二指肠贾第鞭毛虫敏感性适中，特异性较高。RDTs 的主要价值体现在其快速性，以及在腹泻调查中可用于筛查。

Translated from English version into Chinese by Xin-Yu Feng, edited by Pin Yang

CHINESE CENTER FOR DISEASE CONTROL AND PREVENTION
NATIONAL INSTITUTE OF PARASITIC DISEASES

Réalisation de trois tests de diagnostic rapides pour la détection de *Cryptosporidium* spp. et *Giardia duodenalis* chez les enfants souffrant de malnutrition aiguë sévère et de diarrhée.

Joseph Bitilinyu-Bangoh, Wieger Voskuijl, Johnstone Thitiri, Sandra Menting, Nienke Verhaar, Laura Mwalekwa, Daisy B. de Jong, Merlin van Loenenen, Petra F. Mens, James A. Berkley, Robert H.J. Bandsma, et Henk D. F. H. Schallig

Résumé

Contexte: Il existe un besoin important d'outils de diagnostic précis pour les *Cryptosporidium* spp. et *Giardia duodenalis* dans les pays aux ressources limitées où les maladies diarrhéiques causées par ces parasites sont souvent répandues. La présente étude a évalué la performance diagnostique de trois tests de diagnostic rapide (TDR) disponibles sur le marché qui détectent la présence d'antigènes fécaux aux infections au *Cryptosporidium* spp. et/ou *G. duodénalis* dans des échantillons de selles d'enfants admis pour malnutrition aiguë sévère (MAS) et diarrhée. Une PCR multiplex reconnue a été utilisée comme test de référence.

Méthodes: Des échantillons de selles d'enfants atteints de MAS et de diarrhée, recrutés dans un essai clinique randomisé (enregistré sur clinicaltrials.gov/ct2/show/NCT02246296) au Malawi (*n* = 175) et au Kenya (*n* = 120) entre décembre 2014 et décembre 2015, ont été analysés par une PCR multiplexe pour la présence d'ADN des parasites *Cryptosporidium* spp., *G. duodénalis* ou *Entamoeba histolytica* . Les échantillons positifs de *Cryptosporidium*- ont été typés à l'aide d'une analyse de polymorphisme de longueur des fragments de restriction. Un sous-échantillon des échantillons de selles (*n* = 236) a été utilisés avec trois TDR différents lors des tests. L'exactitude diagnostique des tests à l'étude a été évaluée à partir des résultats de la PCR comme étalon de référence à l'aide du logiciel MedCalc. Le test du chi carré de Pearson et le test exact de Fisher ont été utilisés pour déterminer la différence (significative) entre le nombre de cas de cryptosporidiose ou de giardiase détectés par PCR au Malawi et au Kenya. La précision diagnostique globale de chaque TDR a été calculée en traçant une courbe caractéristique de la performance d'un test (ROC) pour chaque test afin de déterminer l'aire sous la courbe (AUC) à l'aide d'un logiciel SPSS8.
Résultats: La prévalence de *Cryptosporidium* spp. par PCR était de 20,0 % et 21,7 % respectivement au Malawi et au Kenya, principalement *C. hominis. La prévalence de G. duodenalis* était de 23,4 % et 5,8 % au Malawi et au Kenya respectivement. *E. histolytica* n'a pas été détectée par PCR. Les tests TDR ont suivi le même schéma de prévalence. Les résultats du TDR variaient entre 42,9 % et 76,9 % pour la cryptosporidiose et de 48,2 % à 85,7 % pour *G. duodenalis* . Les spécificités diagnostiques des TDR variaient entre 88,4 % et 100 % pour le *Cryptosporidium* spp. et de 91,2 % à 99,2 % pour les infections de *G. duodénalis* . Compte tenu de la superficie estimée sous la courbe (SSC), tous les tests évalués avaient une précision diagnostique globale acceptable (> 0,7), à l'exception d'un TDR pour *Cryptosporidium* spp. au Malawi.
Conclusions: Les trois TDR évalués pour le *Cryptosporidium* spp. et le *Giardia duodenalis* dans cette étude ont une sensibilité moyenne, mais une spécificité diagnostique suffisante. La principale valeur des TDR réside dans leur rapidité et leur utilité en tant que tests de dépistage dans les enquêtes sur la diarrhée.

Translated from English version into French by Caroline Giguère, revised by Emilie Rigault Fourcadier, through

TRANSLATORS
WITHOUT BORDERS

Проведение трех быстрых диагностических тестов для обнаружения *Cryptosporidium* spp. и *Giardia duodenalis* у детей с тяжелой острой недостаточностью питания и диареeй

Джозеф Битилиню-Банго, Вигер Воскуайл, Джонстон Титири, Сандра Ментинг, Ниенке Верхар, Лаура Мвалеква, Дейзи Б. де Йонг, Мерлин ван Лунен, Петра Ф. Менс, Джеймс А. Беркли, Роберт Х. Дж. Бандсма и Хэнк Д. Ф. Х. Схаллиг

Резюме

Предпосылки: существует значительная потребность в точных инструментах для диагностирования *Cryptosporidium* spp. и инфекций *Giardia duodenalis* в странах с ограниченными ресурсами, где часто распространены диарейные заболевания, вызываемые этими паразитами. В настоящем исследовании оценивалась диагностическая эффективность трех имеющихся в продаже экспресс-тестов (RDT), основанных на обнаружении фекальных антигенов для *Cryptosporidium* spp. и / или инфекции *G. duodenalis* в образцах стула детей, поступивших с тяжелой острой недостаточностью питания (SAM) и диареей. Установленный сложный ПЦР анализ был использован в качестве контрольного теста.

Методы: образцы стула от детей с SAM и диареей, которые были включены в рандомизированное контролируемое исследование (зарегистрированное по адресу: Clinicaltrials.gov/ct2/show/NCT02246296) в Малави (*n* = 175) и Кении (*n* = 120) в период с декабря 2014 года по декабрь 2015 года, были проанализированы методом сложного ПЦР анализа на наличие ДНК паразита *Cryptosporidium* spp., *G. duodenalis* или *Entamoeba histolytica*. Криптоспоридиум-положительные образцы были классифицированы с использованием анализа полиморфизма длины рестрикционных фрагментов. Подвыборка образцов кала (*n* = 236) была использована для тестирования с тремя различными RDT. Точность диагностики на основе данных тестов оценивалась при использовании в качестве эталонного стандарта результатов ПЦР с помощью программного обеспечения MedCalc. Для выявления (значительного) различия между числом случаев криптоспоридиоза или гиардиаза, обнаруженных методом ПЦР в Малави и Кении, использовались критерий хи-квадрат Пирсона и точный критерий Фишера Общая диагностическая точность каждого RDT рассчитывалась путем построения кривой рабочей характеристики приемника (ROC) для каждого теста и определения площади под кривой (AUC) с использованием программного обеспечения SPSS8.

Результаты: распространенность *Cryptosporidium* spp., по ПЦР, составила 20,0% и 21,7% в Малави и Кении, соответственно, в основном, это был *C. hominis*. Распространенность *G. duodenalis* в Малави и Кении составила 23,4% и 5,8% соответственно. *E. histolytica* не была обнаружена с помощью ПЦР. Тестирование по RDT выявило ту же схему распространенности. Чувствительность RDT варьировала, для криптоспоридиоза, от 42,9% до 76,9% и, для *G. Duodenalis,* от 48,2% до 85,7%. Специфичность RDT варьировала от 88,4% до 100% для *Cryptosporidium spp.* и от 91,2% до 99,2% для инфекций *G. duodenalis.* Судя по значениям расчетной площади под кривой (AUC) все оцениваемые тесты имели приемлемую общую диагностическую точность (> 0,7) (за исключением одного RDT для *Cryptosporidium spp.* в Малави).

Выводы*:* все три RDT для *Cryptosporidium* spp. и *Giardia duodenalis,* оцененные в этом исследовании, обладают умеренной чувствительностью, но достаточной специфичностью. Основная ценность RTD заключается в их быстроте и полезности для скриниинговых анализов при обследованиях на диарею.

Translated from English version into Russian by Maria Petrenko, revised by Alexander Somin, through

TRANSLATORS WITHOUT BORDERS

La precisión de tres pruebas de diagnóstico rápido para la detección de *Cryptosporidium* spp. y *Giardia duodenalis* en niños con desnutrición aguda grave y diarrea.

Joseph Bitilinyu-Bangoh, Wieger Voskuijil, Johnstone Thitiri, Sandra Menting, Nienke Verhaar, Laura Mwalekwa, Daisy B. de Jong, Merlin van Loenen, Petra F. mens, Jmes A Berkley, Robert H. J. Bandsma y Henk D. H. Schallig

Resumen

Antecedentes: existe una importante necesidad de contar con herramientas de diagnóstico precisas para las infecciones por *Cryptosporidium* spp. y *Giardia duodenalis* en países de recursos limitados donde la diarrea causada por estos parásitos es frecuente. El presente estudio evaluó la precisión de tres pruebas de diagnóstico rápido disponibles en el mercado (RDT, por sus siglas en inglés) basadas en la detección del antígeno fecal para infecciones por *Cryptosporidium* spp. o *G. duodenalis* en muestras de heces obtenidas de niños ingresados con desnutrición aguda grave (SAM, por sus siglas en inglés) y diarrea. Como prueba de referencia se utilizó una PCR múltiple establecida.

Métodos: muestras de heces de niños con SAM y diarrea incluidos en un ensayo controlado aleatorio (registrado en clinicaltrials.gov/ct2/show/NCT2246296) en Malawi (*n*= 175) y Kenia (*n* = 120) entre diciembre de 2014 y diciembre de 2015 se analizaron por PCR múltiple para detectar la presencia de ADN de los parásitos *Cryptosporidium* spp., *G. duodenalis* o *Entamoeba histolytica*. Se tipificaron las especies de las muestras positivas para *Cryptosporidium* utilizando el análisis de fragmentos de restricción de longitud polimórfica. Se utilizó una submuestra de materia fecal (*n* = 236) para evaluarla con tres RDT distintas. La precisión del diagnóstico de las pruebas objeto de la evaluación se analizó usando los resultados de la PCR como norma de referencia y el programa MedCalc. Las prueba de chi cuadrado de Pearson y la prueba exacta de Fisher se usaron para determinar la existencia de una diferencia (significativa) entre el número de casos de criptosporidiosis o giardiasis detectados por PCR en Malawi y Kenia. La precisión general del diagnóstico de cada RDT se calculó trazando una curva de la característica operativa del receptor (ROC, por sus siglas en inglés) para cada prueba y para determinar el área bajo la curva (AUC, por sus siglas en inglés) usando el programa SPSS8.
Resultados: la prevalencia de *Cryptosporidium* spp. por PCR fue del 20,0 % y del 21,7% en Malawi y Kenia respectivamente, en su mayoría *C. hominis. La prevalencia de G. duodenalis* fue del 23,4% y 5,8% en Malawi y Kenia respectivamente. No se detectó *E. histolyca* por medio de PCR. Las RDT siguieron el mismo patrón de prevalencia. Las sensibilidades de las RDT para criptosporidiosis variaron entre el 42,9% y el 79,9% y para *G. duodenalis* entre el 48,2% y el 85,7%. Las especificidades de las RDT variaron entre el 88,4% y el 100% para las infecciones por *Cryptosporidium* spp. y entre el 91,2% y el 99,2% para aquellas por *G. duodenalis*. Sobre la base de los valores estimados del área bajo la curva (AUC), todas las pruebas evaluadas obtuvieron una precisión de diagnóstico general aceptable (> 0,7), con la excepción de una prueba de diagnóstico rápido para *Cryptosporidium* spp. en Malawi.
Conclusiones: las tres pruebas de diagnóstico rápido para *Cryptosporidium* spp. y *Giardia duodenalis* evaluadas en este estudio tienen una sensibilidad moderada, pero suficiente especificidad. El principal valor de las RDT radica en su rapidez y su utilidad como pruebas de cribado en estudios sobre la diarrea.

Translated from English version into Spanish by Patricia Martinez, revised by Mayra Léon, through

TRANSLATORS
WITHOUT BORDERS
